# From Infancy to Aging: Precise Brain Age Estimation via Hybrid CoTResNet3D and CrossViT Models on T1-Weighted Imaging

**DOI:** 10.3390/bioengineering13030315

**Published:** 2026-03-09

**Authors:** Xinyu Zhu, Shen Sun, Hongjian Gao, Yutong Wu, Zhenrong Fu, Lan Lin

**Affiliations:** 1 Department of Biomedical Engineering, College of Chemistry and Life Science, Beijing University of Technology, Beijing 100124, China; zhuxyu@emails.bjut.edu.cn (X.Z.); sunshen@bjut.edu.cn (S.S.); gaohongjian@bjut.edu.cn (H.G.); 2 Intelligent Physiological Measurement and Clinical Translation, Beijing International Base for Scientific and Technological Cooperation, Beijing University of Technology, Beijing 100124, China; 3 Key Laboratory of Adolescent CyberPsychology and Behavior (CCNU), Ministry of Education, Wuhan 430079, China; yutong.wu@mails.ccnu.edu.cn; 4 Key Laboratory of Human Development and Mental Health of Hubei Province, School of Psychology, Central China Normal University, Wuhan 430079, China

**Keywords:** brain age, T1-weighted MRI, CNN, Transformer, cross-center generalization

## Abstract

Accurate estimation of brain age from structural magnetic resonance imaging (MRI) serves as a vital biomarker for quantifying individual neurobiological aging and identifying risks for neurological disorders. However, developing robust models that generalize across the entire lifespan (from infancy to aging) remains challenging due to heterogeneous maturation/degeneration patterns, limited cross-center generalizability, and insufficient temporal reliability evaluation. To address these limitations, we curated a large-scale, multi-center T1-weighted MRI dataset across 27 public cohorts. Of these, 22,271 scans from 17 cohorts (aged 0–96 years) formed the primary foundation for model development, complemented by 10 additional cohorts utilized for independent multi-center evaluation and robustness testing. We propose ResNet-CrossViT, a novel hybrid architecture that synergistically combines a 3D Contextual Transformer-ResNet (CoTResNet3D) backbone for enriched local feature extraction and a CrossVision Transformer (CrossViT) module for cross-scale global dependency modeling. The model was rigorously evaluated on an internal test set, an unseen external dataset for cross-center validation, a longitudinal dataset for assessing temporal consistency, and a test–retest dataset for measuring reproducibility. On the internal test set, ResNet-CrossViT achieved a mean absolute error (MAE) of 2.72 years and a maximal MAE (mMAE) of 5.10 years, demonstrating marked performance improvements, particularly within the challenging adolescent cohort. The model maintained strong generalization on the unseen dataset (MAE = 4.19 years) and exhibited superior longitudinal consistency (Mean Absolute Difference Error, MAdE = 3.68) and excellent test–retest reliability (Intraclass Correlation Coefficient, ICC = 0.994). By integrating a large-scale, heterogeneous lifespan dataset with a hybrid architecture that effectively captures both local structural details and global long-range interactions, our study provides a precise, generalizable, and reliable framework for brain age estimation.

## 1. Introduction

As individuals age, their susceptibility to a wide range of diseases increases, with neurological disorders drawing significant attention in recent years [1]. The brain, the most complex organ in the human body, undergoes a progressive irreversible aging process that manifests in both structural and functional decline. This decline is closely linked to conditions such as Alzheimer’s disease (AD), depression, and bipolar disorder [2,3,4]. Historically, the ability to assess brain aging has been constrained by technological limitations, but recent advances in neuroimaging techniques have dramatically transformed our understanding of brain aging [5,6]. Rapid progress in neuroimaging, including magnetic resonance imaging (MRI), computed tomography (CT), and positron emission tomography (PET), have paved the way for more precise analyses of brain structure and function. These advancements have been instrumental in developing the concept of “brain age”, a measure that quantifies the physiological state and age-related changes in the brain. Brain age is estimated by analyzing imaging biomarkers such as gray matter volume, white matter integrity, and functional connectivity, in combination with machine learning, to quantify the brain’s physiological state and degree of aging [6]. The gap between predicted brain age and an actual chronological age, often termed the brain age gap (BAG), is a critical indicator of brain health [7]. If the predicted brain age is younger than the individual’s chronological age, this typically suggests a brain that is structurally youthful and exhibits better functional capacity. Conversely, a brain age that is older than the chronological age may signal accelerated aging, which could predispose individuals to neurological disorders [8] or indicate underlying pathologies that have not yet manifested clinically [9,10]. As a result, brain age prediction holds important scientific and clinical value for advancing neuroscience research, facilitating early disease screening, and enabling personalized health management strategies.

In the early stages of brain age prediction, studies primarily relied on traditional machine learning (ML) approaches, such as support vector machines (SVM), Lasso regression, and XGBoost, to estimate brain age from neuroimaging data. For instance, Pojoga and colleagues developed a framework for predicting brain age from functional connectivity matrices using a multilayer perceptron (MLP) as a meta-learner, integrating multiple regressors to reduce bias and improve accuracy, achieving an average Root Mean Square Error (RMSE) of about 1.6 years [11]. Similarly, Costa and colleagues introduced a shallow ensemble combining support vector regression, linear regression, and Gaussian process regression, with hyperparameters tuned by genetic algorithms and grid search, reaching a mean absolute error (MAE) of 3.76 years in the PAC 2019 challenge [12]. A recent study by Xiong et al. [13] compared six machine learning models for brain age prediction across six image modalities in middle-aged and older adults, highlighting Lasso as a promising ML algorithm for brain age prediction. Despite their popularity, traditional machine learning methods struggle with the complexity and high dimensionality of neuroimaging data. Reliance on manually engineered features limits their ability to capture nonlinear relationships [14], and their performance is highly dependent on sample size, requiring large, diverse datasets for effective generalization.

With the increasing scale of data and complexity of models, deep learning (DL) has become the dominant approach for brain age prediction due to its ability to automatically learn hierarchical representations and extract relevant features. Sajjadi et al. proposed a multi-task self-supervised framework combining brain age prediction with an image-rotation classification task and Distillation with No Labels (DINO) pretraining, achieving an MAE of 3.27 years on the Alzheimer’s Disease Neuroimaging Initiative (ADNI) dataset [15]. Amoroso et al. introduced a hybrid model that integrates multilayer complex networks and deep neural networks, achieving an MAE of 4.7 years across five public datasets [16]. Usui et al. adapted a ResNet-50 for brain age prediction, obtaining an MAE of 5.25 years over samples aged 5 to 79 [17], while Dartora and colleagues developed a lightweight 3D ResNet model, using T1-weighted MRI (T1w MRI) volumes that were rigidly registered to Montreal Neurological Institute (MNI) space as input. Four training configurations (CNN1–CNN4) were constructed on the same backbone to systematically compare the effects of hold-out versus cross-validation strategies, the inclusion of external cohorts, and the use of skull-stripped images on model performance and generalizability. Across multiple datasets, the model achieved MAE in the range of approximately 2.7 to 3.1 years [18]. Jahanshiri et al. combined six 2D CNNs (Convolutional Neural Networks) with two 3D CNNs, producing an MAE of 3.57 years on the BANK dataset [19]. Building on a lightweight fully convolutional network reported by Peng et al. that surpassed deeper CNNs on brain age and sex prediction [20], Eltashani and colleagues further proposed a compact 3D CNN with transfer learning, achieving an MAE of 3.56 years on 2251 multi-center MRIs and reducing training time by 50% [21].

While CNNs are highly effective at extracting local features, they face limitations in modeling global dependencies among distant brain regions. Their inherently local receptive fields restrict their ability to capture long-range interactions and whole-brain structural relationships, which are crucial for accurate brain age prediction. The Transformer architecture, introduced by Vaswani et al. in 2017 [22], addressed these limitations. The core self-attention mechanism in Transformers allows the model to efficiently capture long-range dependencies in sequences, overcoming the locality constraints of CNNs. As Transformers have been adapted for neuroimaging, their capacity to model whole-brain spatial dependencies, integrate information across modalities has significantly enhanced the performance of brain age prediction models.

In recent years, Transformer-based models for brain age prediction have rapidly advanced, demonstrating notable strengths in capturing long-range dependencies and integrating multimodal information. For instance, Cai et al. [23] proposed a geometric deep learning framework based on a graph Transformer, which jointly models structural Magnetic Resonance Imaging (sMRI) and diffusion tensor imaging (DTI), showing that Transformers can effectively capture long-range multimodal interactions across brain regions in a graph representation. Tian et al. [24] introduced Transformer^3^, which constructs Transformer modules along sample, region, and temporal dimensions, thereby simultaneously modeling inter-individual variability, spatial dependencies, and temporal dynamics in functional Magnetic Resonance Imaging (fMRI). Their framework demonstrated strong high-dimensional spatiotemporal modeling capability on the Nathan Kline Institute Rockland Sample (NKI-RS dataset). In another direction, Dhinagar and colleagues [25] employed a Transformer-based Masked Autoencoder pretrained on large-scale UK Biobank (UKB) MRI data and achieved an MAE of 4.09 years; however, the downstream model still primarily relies on global token representations, limiting its sensitivity to fine-grained structural variations. In the context of three-dimensional MRI, Jun et al. [26] proposed the Medical Transformer, which decomposes 3D MRI volumes into multi-view 2D sequences and combines CNN-based local feature extraction with Transformer-based global relational modeling, achieving an MAE of 3.49 years on the ADNI dataset. Liu et al. [27] developed the Sparse Transformer Association Analysis (STAA), which integrates convolutional autoencoders with a sparse Transformer to model imaging—genetic associations, reaching an MAE of 3.15 years on the UKB dataset. Yang et al. [28] further introduced the Global—Local Dependency Network (GLDN), incorporating a Successive Permuted Transformer to enhance long-range 3D dependency modeling while leveraging CNNs to preserve local structural detail, achieving an MAE of 2.91 years across five large-scale datasets.

Collectively, these studies underscore the strong modeling capability of Transformer architectures in brain age prediction. Their self-attention mechanisms enable the establishment of global dependencies across patches, slices, or brain-region nodes, allowing the models to capture complex structural patterns, integrate multimodal information, and process multi-view representations. However, despite these notable advantages, the broader applicability of Transformer-based models in brain age prediction is constrained by a combination of intrinsic architectural limitations and shortcomings in prevailing research practices. Primarily, the architectural design of many existing Transformer models presents two fundamental hurdles. First, many existing methods rely on 2D slices or 3D patches as inputs, which restricts their ability to fully exploit whole-brain 3D structural information and the coordinated interactions across distant regions. Second, due to the absence of the local inductive bias inherent in convolutional networks, Transformers often struggle to capture fine-grained anatomical details, such as sulcal boundaries, cortical thickness, and subtle morphological variations—that are essential for accurate brain age estimation [29,30,31]. Consequently, these architectural constraints are compounded by common limitations in model development and evaluation paradigms. A significant challenge lies in the limited generalization of current Transformer-based models across heterogeneous datasets and populations. Most models are evaluated on datasets that closely resemble their training distribution, resulting in strong in-sample performance but substantial degradation when transferred to datasets with different demographic compositions, imaging protocols, or scanner characteristics. Furthermore, exacerbating the generalization issue, many cohorts used for training and evaluation cover relatively narrow age ranges, overlooking the substantial variability of brain structure across childhood, adulthood, and older age. This limitation not only undermines the stability of the models but also introduces potential biases that may affect their performance across the entire lifespan.

To address these limitations, our study prioritizes generalizability by constructing a comprehensive neuroimaging resource that spans the lifespan, coupled with rigorous evaluations across multi-center, multi-scanner, and multi-batch conditions. This approach not only facilitates large-scale model training but also extends evaluation metrics beyond overall accuracy to include age-stratified errors, test–retest reliability, and multi-session consistency. These measures offer a more nuanced assessment of model transferability and robustness across the lifespan stages. Building on this foundation, we introduce a novel ResNet-CrossViT hybrid architecture that leverages the complementary strengths of CNNs and Transformers. Central to this design is the integration of a Contextual Transformer (CoT) module [32], which embeds self-attention within a convolutional framework, along with a multi-scale cross-patch attention mechanism inspired by Cross-Attention Multi-Scale Vision Transformer (CrossViT) [33]. This architecture enables joint modeling of local and global information in an efficient manner. By combining fine-grained structural details with long-range semantic dependencies, the hybrid model mitigates the limitations of pure CNN or Transformer models, thereby enhancing convergence and generalization in high-dimensional neuroimaging tasks. In summary, through the creation of a heterogeneous lifespan imaging resource and the development of an enhanced hybrid modeling framework, this study provides a more robust and scalable solution that more effectively captures complex structural patterns in brain images and improves generalization across populations, scanners, and imaging environments.

## 2. Materials and Methods

### 2.1. Datasets

To construct the database, we began with a systematic survey of publicly available datasets suitable for brain-age prediction, categorizing them into open-access and non-open-access resources. In alignment with our focus on reproducibility and open availability, we excluded non-open-access datasets. Given our goal was to create a large, lifespan-spanning neuroimaging database, and based on prior research indicating T1w MRI is typically the only sequence consistently available across cohorts exceed 10,000 cases [34,35], we retained only datasets containing T1w MRI and removed those lacking this sequence. In addition, to reduce confounding and bias introduced by differences in sample age structure, we aligned the age distributions across datasets. Following this stringent screening process, 27 datasets met the inclusion criteria: (1) healthy participants, (2) availability of T1w MRI for all participants, and (3) complete demographic information, including age even if approximate and sex. Importantly, all experiments were conducted on real-world T1-weighted structural MRI acquired from heterogeneous multi-cohort datasets across the lifespan. To enhance the generalizability and robustness of the brain-age model, we intentionally retained diversity in acquisition parameters, including scanner vendors, resolutions, noise levels, and imaging protocols. This diversity minimizes the risk of the model being biased toward any specific imaging condition.

In terms of experimental design, the included datasets contributed subjects to two major subsets: an internal dataset for model development and evaluation subsets for performance assessment. Among the 27 datasets that met the inclusion criteria, 17 datasets contributed subjects to the internal dataset used for model training, validation, and internal testing, while the remaining datasets contributed subjects exclusively to evaluation subsets. The internal dataset comprised subjects from these 17 databases and was randomly divided into training (70%), validation (15%), and internal test (15%) sets. To comprehensively evaluate model stability and generalizability, evaluation was performed using three complementary subsets defined at the subject level. The Unseen Dataset consisted of subjects from independent cohorts that were not used for model training, validation, or internal testing. These cohorts were entirely excluded from model development and were used to evaluate model generalization to previously unseen datasets. The Longitudinal Dataset included repeated scans from the same participants at multiple time points to evaluate temporal consistency of brain age estimation. The Test–Retest Dataset comprised repeated scans acquired over short intervals to assess measurement reliability and repeatability. Importantly, for cohorts contributing to multiple subsets, subject assignment was strictly non-overlapping. Longitudinal and test–retest scans used for evaluation were excluded from the internal training, validation, and test sets to prevent data leakage. All MRI underwent rigorous visual quality control, and images with pronounced motion artifacts or signal abnormalities were excluded. The final cohort of healthy participants covered ages 0 to 96 years, ensuring broad lifespan coverage. The training and validation sets were used for model learning and parameter optimization, whereas the internal and unseen external test sets were used for performance assessment and comparison.

#### 2.1.1. Datasets Description

This study provides a systematic curation of key database attributes, including participant age ranges, sample sizes of healthy controls, sex ratios, country distributions, scanner types, and magnetic field strengths. We also explicitly annotate the categorization of each dataset within this study to help readers quickly identify its role and intended use. Specific details of the datasets are presented in Table 1.

**Figure 1:**
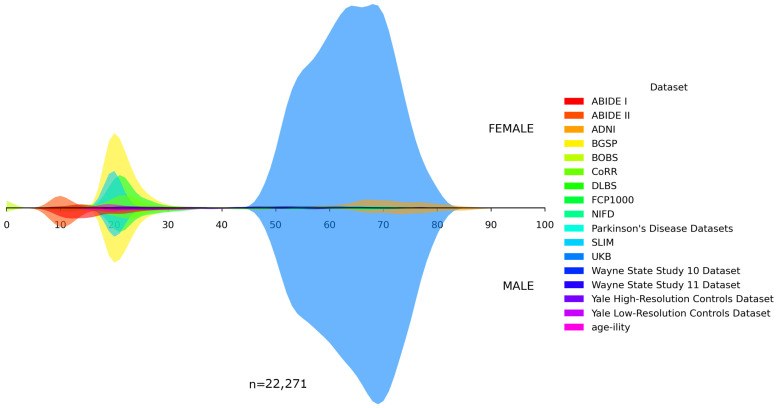
Sex-stratified age distribution of the total internal dataset (n = 22,271).

**Figure 2:**
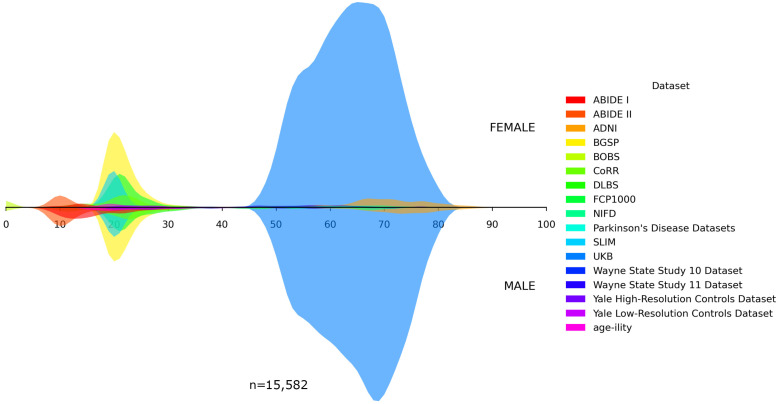
Age distribution of the internal training set (n = 15,582).

**Figure 3:**
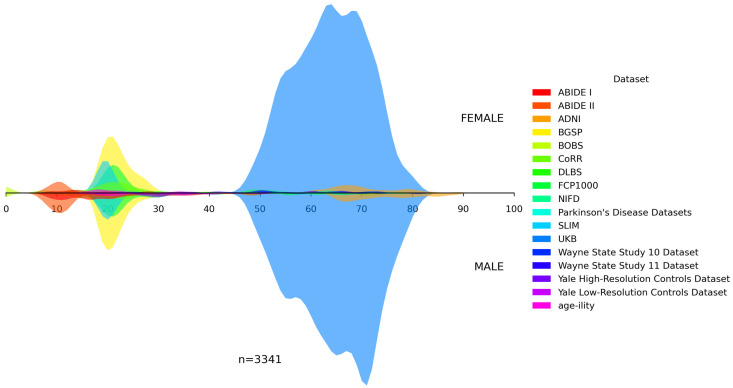
Age distribution of participants of the internal validation set (n = 3341).

**Figure 4:**
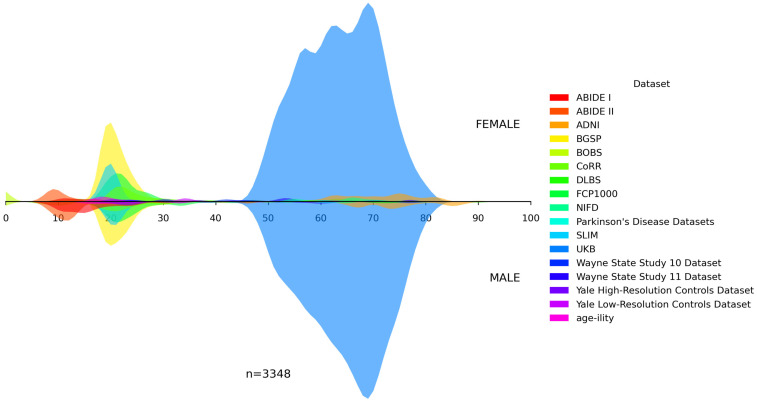
Age distribution of the internal test set (n = 3348).

**Figure 5:**
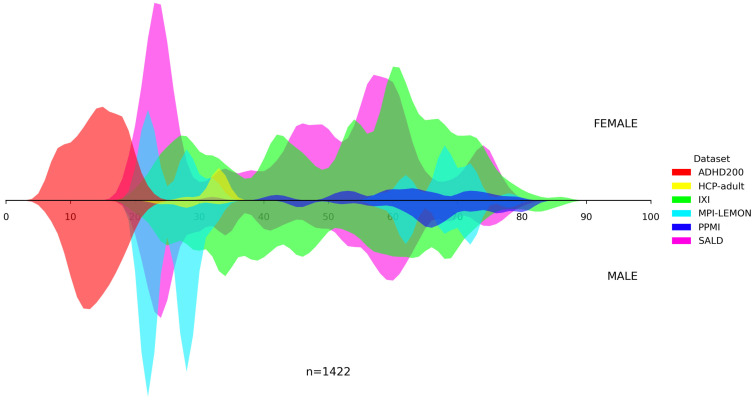
Age distribution of the independent unseen dataset (n = 1422).

In addition, we visualized the age distributions for both the internal and the unseen dataset. Figure 1, Figure 2, Figure 3, Figure 4 and Figure 5 illustrated these distributions, including a detailed breakdown of the training, validation, and test subsets within the internal dataset. The training, validation, and test subsets were generated through randomized partitioning, ensuring that the demographic characteristics and age–sex distributions remain consistent with the original internal dataset.

#### 2.1.2. Data Preprocessing

The preprocessing of T1w MRI was performed using the CAT12 toolbox (Computational Anatomy Toolbox for SPM, https://neuro-jena.github.io/cat/) within the SPM12 (Statistical Parametric Mapping, https://www.fil.ion.ucl.ac.uk/spm/software/) environment. The primary step involved tissue segmentation and spatial normalization, both conducted through the CAT12 “segment” module. During the normalization process, each participant’s native structural volume was registered to a common atlas space to ensure spatial consistency across all subjects.

To ensure the quality and reliability of the data, we implemented a strict quality control (QC) procedure. Initially, the CAT12 image quality score (IQR) was used for screening. Data samples with IQR below 75 were excluded from the analysis. For samples with an IQR score of 75 or higher, we conducted a manual visual inspection to further filter out images exhibiting severe motion artifacts or signal abnormalities. This two-stage QC process safeguarded the accuracy of tissue segmentation and normalization.

After preprocessing, we extracted gray matter density maps (GMDM) with isotropic 1 × 1 × 1 mm^3^ voxels and image dimensions of 169 × 205 × 169 for each volume. For GMDM, modulation was applied to account for inter-individual differences in brain volume and morphology, yielding volume-normalized images suitable for downstream analysis. To further optimize model training and reduce computational burden, we downsampled the GMDM to a lower resolution of 84 × 102 × 84 voxels using trilinear interpolation with isotropic 2 × 2 × 2 mm^3^ resolution. This step preserved key anatomical features while significantly reducing data volume and computational complexity.

### 2.2. Performance Metrics

To comprehensively evaluate model performance across diverse application scenarios, we partitioned the dataset based on functional utility into four subsets: an internal test set, an unseen dataset, a longitudinal dataset, and a test–retest dataset. Subsequently, for each subset, we employed task-appropriate evaluation metrics to quantify (i) predictive accuracy and robustness, (ii) longitudinal consistency, and (iii) reproducibility.

#### 2.2.1. Accuracy and Robustness

For the internal test set and the unseen dataset, we assessed predictive accuracy and robustness through a comprehensive suite of quantitative metric. The primary measure used for prediction accuracy was the MAE, which quantified the average absolute deviation between the predicted age and the chronological (true) age:

(1) $$MAE=\frac{1}{N}\sum_{i=1}^{N}\left|{\hat{y}}_{i}-y_{i}\right|$$

where $y_{i}$ denotes the true age of the i-th subject, ${\hat{y}}_{i}$ denotes the predicted age of the i-th subject, and N is the total number of subjects. A smaller MAE indicates higher overall prediction accuracy.

To complement this, we used the mean error (ME) to quantify systematic bias, specifically whether the model consistently tends to overestimate or underestimate age:

(2) $$ME=\frac{1}{N}\sum_{i=1}^{N}\left({\hat{y}}_{i}-y_{i}\right)$$

Here, an ME close to 0 indicates the absence of systematic bias; whereas positive and negative values signify overall tendencies to overestimate and underestimate, respectively.

To evaluate performance stability across different age ranges, we further employed the maximal MAE (mMAE). This metric stratifies subjects into age bins according to their chronological age, computes the MAE within each bin, and then identifies the maximum MAE across all bins. Formally, given *K* age intervals (bins), and the *k*-th bin is $\left[b_{k},b_{k+1}\right)$. Let $I_{k}=\{i|y_{i}\in [b_{k},b_{k+1})\}$ denote the index set of samples in the *k*-th bin, and let $N_{k}=\left|I_{k}\right|$ be the number of samples in that bin. The MAE for the *k*-th bin is expressed as:

(3) $${MAE}_{k}=\frac{1}{N_{k}}\sum_{i\in I_{k}}\left|{\hat{y}}_{i}-y_{i}\right|$$

The final mMAE is consequently defined as:

(4) $$mMAE=\underset{k=1,\ldots ,K}{max}{MAE}_{k}$$

Here, *K* is the number of age bins; $b_{k}$ and $b_{k+1}$ are the left and right boundaries of the *k*-th bin, respectively; $I_{k}$ is the set of sample indices within that bin; $N_{k}$ is the corresponding sample count; and ${MAE}_{k}$ is the MAE within that bin. The mMAE reflects the prediction error in the worst-performing age range. A small discrepancy between the mMAE and the global MAE suggests that model performance is equitably distributed and robust across age groups. In the specific implementation of this study, the chronological ages of subjects in the test set ranged from 0 to 96 years. We grouped subjects into age intervals of 10 years each, and for the terminal interval with a span of less than 10 years, a separate group was formed according to the actual age range. In total, 10 age intervals were defined: [0, 10), [10, 20), …, [80, 90), and [90, 100). All age intervals followed a left-closed, right-open convention. The MAE was computed separately for each age interval, and the maximum MAE among the 10 intervals was reported as the final mMAE metric. Because the test set contained only n = 2 subjects in the [90, 96) age interval, the mMAE was potentially inflated by outliers. To ensure objective assessment of worst-case performance, we excluded this small-sample bin from mMAE calculation and reported the maximum MAE across remaining age bins as the final mMAE.

In addition, the Pearson correlation coefficient (*r*) was used to quantify the strength of the linear association between the predicted ages and the true ages, while the coefficient of determination (*R*^2^) was employed to measure the proportion of variance in the true ages that can be explained by the model predictions:

(5) $$r=\frac{\sum_{i=1}^{N}\left(y_{i}-\overset{‾}{y}\right)\left({\hat{y}}_{i}-\bar{\hat{y}}\right)}{\sqrt{\sum_{i=1}^{N}{\left(y_{i}-\overset{‾}{y}\right)}^{2}}\sqrt{\sum_{i=1}^{N}{\left({\hat{y}}_{i}-\bar{\hat{y}}\right)}^{2}}}$$

(6) $$R^{2}=1-\frac{\sum_{i=1}^{N}{\left(y_{i}-{\hat{y}}_{i}\right)}^{2}}{\sum_{i=1}^{N}{\left(y_{i}-\overset{‾}{y}\right)}^{2}}$$

Here, $\bar{y}$ denotes the mean of the true ages and $\bar{\hat{y}}$ denotes the mean of the predicted ages. In general, values of |r| closer to 1 indicate a stronger linear correlation, whereas larger values of *R*^2^ signify superior goodness of fit and a higher explanatory power of the model with respect to the variability of chronological age.

#### 2.2.2. Consistency

For the longitudinal dataset, we adopted the following metrics to evaluate model consistency. For healthy participants, the predicted increase in brain age across consecutive follow-up scans should ideally match the actual elapsed time. To assess this, for the *i*-th subject with two scans—baseline (B) and follow-up (F)—we quantified the discrepancy between the predicted age change and the true chronological interval. The definitions are as follows:

True change: $\Delta y_{i}=y_{i2}-y_{i1}$

Predicted change: $\Delta {\hat{y}}_{i}={\hat{y}}_{i2}-{\hat{y}}_{i1}$

Change error: $\delta_{i}=\Delta {\hat{y}}_{i}-\Delta y_{i}$

We further defined the Mean Difference Error (MdE), Mean Absolute Difference Error (MAdE), and Maximal Mean Absolute Difference Error (mMAdE) as follows:

(7) $$MdE=\frac{1}{N}\sum_{i=1}^{N}\left[\left({\hat{y}}_{i2}-{\hat{y}}_{i1}\right)-\left(y_{i2}-y_{i1}\right)\right]=\frac{1}{N}\sum_{i=1}^{N}\delta_{i}$$

(8) $$MAdE=\frac{1}{N}\sum_{i=1}^{N}\left|\left({\hat{y}}_{i2}-{\hat{y}}_{i1}\right)-\left(y_{i2}-y_{i1}\right)\right|=\frac{1}{N}\sum_{i=1}^{N}\left|\delta_{i}\right|$$

(9) $${MAdE}_{k}=\frac{1}{N_{k}}\sum_{i\in I_{k}}\left|\delta_{i}\right|$$

(10) $$mMAdE=\underset{k=1,\ldots ,K}{max}MAdE_{k}$$

where $y_{i1}$ and $y_{i2}$ denote the true chronological ages of subject *i* at B and F, respectively; ${\hat{y}}_{i1}$ and ${\hat{y}}_{i2}$ denote the corresponding predicted brain ages; $\Delta y_{i}$ represents the true F interval (in years); $\Delta {\hat{y}}_{i}$ represents the predicted F interval (in years); $\delta_{i}$ is the consistency error (predicted change minus true change). Physically, MdE values closer to 0 indicate smaller systematic bias, whereas smaller MAdE and mMAdE values indicate superior longitudinal consistency and higher reliability in measuring individual aging trajectories.

#### 2.2.3. Reproducibility

For the test–retest dataset, we used the following metrics to evaluate reproducibility. A model is considered highly reproducible if it produces consistent predictions for the same subject across multiple repeated scans. For each subject *i*, let ${\hat{y}}_{i1}$ and ${\hat{y}}_{i2}$ denote the predicted brain ages from the first and second scans, respectively. We first compute the within-subject standard deviation of the two predictions:

(11) $$\sigma_{i}=SD\left({\hat{y}}_{i1},{\hat{y}}_{i2}\right)$$

We then average this quantity across all subjects with paired test–retest scans:

(12) $$\sigma \left({\hat{y}}_{scan}^{\prime }\right)=\frac{1}{M}\sum_{i=1}^{M}\sigma_{i}$$

where *M* is the number of subjects with paired scans. A smaller $\sigma \left({\hat{y}}_{scan}^{\prime }\right)$ indicates minimal prediction fluctuations for the same subject, reflecting higher predictive stability.

In addition, we use the mean difference *μ*(*d*) to assess whether there is a systematic shift between the two repeated measurements:

(13) $$\mu \left(d\right)=\frac{1}{N}\sum_{i=1}^{N}d_{i=}\frac{1}{N}\sum_{i=1}^{N}{\hat{y}}_{i1}-{\hat{y}}_{i2}$$

Theoretically, *μ*(*d*) close to 0 suggest the absence of systematic bias. A positive *μ*(*d*) (*μ*(*d*) > 0) indicates that predictions from the first scan are, on average, higher than those from the second scan, whereas a negative *μ*(*d*) (*μ*(*d*) < 0) indicates the opposite.

To characterize the dispersion of test–retest differences, we compute the standard deviation of differences *σ*(*d*); smaller values indicate greater stability:

(14) $$\sigma \left(d\right)=SD\left(d_{1},d_{2},\ldots ,d_{N}\right)$$

Finally, the Intraclass Correlation Coefficient (ICC) is employed to quantify both agreement and reliability under repeated measurements. We adopted ICC(3,1) (two-way mixed-effects model, consistency definition, single measurement) to evaluate test–retest reproducibility, defined as:

(15) $$ICC\left(3,1\right)=\frac{MS_{subject}-MS_{error}}{MS_{subject}+\left(k-1\right)MS_{error}}$$

where $MS_{subject}$ is the mean square between subjects, $MS_{error}$ is the residual mean square, and *k* is the number of repeated measurements (here *k* = 2). Note that ICC(3,1) under the consistency definition primarily evaluates whether the relative ranking of subjects’ predictions remains stable across repeated scans, rather than requiring strict numerical equality between the two predicted values. Therefore, even when some test–retest variability exists (*σ*(*d*) ≠ 0), a high ICC still indicates that the model can reliably discriminate individuals by maintaining a stable ordering of predicted brain age. Conventionally, ICC values below 0.5 indicate poor reliability, values between 0.5 and 0.75 indicate moderate reliability, values between 0.75 and 0.90 indicate good reliability, and values above 0.90 indicate excellent reliability.

### 2.3. Model Architecture

This study proposes a hybrid 3D deep learning architecture for brain age prediction. The model comprises two complementary components: an enhanced 3D convolutional backbone (CoTResNet3D) that extracts multi-scale spatial structural features, and a Transformer-based cross-scale attention module that converts features from different scales into token sequences for sequence modeling and mutual fusion. The overarching design rationale is to couple the local pattern recognition efficiency of CNNs with the global long-range dependency modeling of Transformers, thereby deriving feature representations that are both more discriminative and more interpretable for medical imaging data, which simultaneously encode fine-grained anatomical details and whole-brain associations. Although convolution progressively aggregates neighborhood information, its effective receptive field and inductive bias remain predominantly local; in brain aging, however, subtle but distributed anatomical variations across distant regions (e.g., cortical and subcortical structures) jointly contribute to age-related effects. Therefore, the Transformer module explicitly learns global, non-local associations and enables mutual fusion between scales, yielding representations that encode both fine-grained anatomical details and whole-brain structural dependencies.

To fully leverage the potential of the proposed hybrid architecture, we conducted a systematic optimization of the model’s configuration. This process was decoupled into two sequential stages to efficiently navigate the expansive hyperparameter space, and optimization strategies were determined through a comprehensive grid search on the validation set. First, we performed a structural architecture search to establish the optimal backbone and fusion configuration. This stage focused on the fundamental capacity of the model, where we varied the number of residual blocks in the CoTResNet3D stages (ranging from 1 to 3 blocks per stage) and the Transformer layers within the CrossViT modules (selected from {2, 3, 4} layers per branch). Key structural hyperparameters were explored within this phase, including the embedding dimension {256, 512, 1024} and the patch size for tokenization {1, 2, 3}. Based on validation performance, the final architecture was fixed with a 2-block configuration for each CoTResNet3D stage, an embedding dimension of 512, a patch size of 2, and a 3-layer configuration for each CrossViT branch.

In the second stage (optimization strategy search), with the architecture fixed, we refined five core parameters to ensure stable convergence and robust generalization: the peak learning rate (5 × 10^−5^ to 5 × 10^−4^), the warmup duration (0–10 epochs), the drop_path_rate ([0.0, 0.3]), the auxiliary ranking loss weight λ in [0, 0.02], and the loss function formulation (comparing L1, MSE, and Huber loss). The optimal values identified through this search were a peak learning rate of 2 × 10^−4^, a 5-epoch warmup, a drop_path_rate of 0.1, an auxiliary weight λ of 0.005, and the MSE loss function. To ensure reproducibility and a fair comparison, all experiments—including the proposed model and all baseline architectures—were implemented using the same computational framework. All models were developed in Python (v3.10.0) using the PyTorch (v2.0.1) framework with CUDA 11.8 and cuDNN 8.7 support, and executed on a workstation equipped with an Intel Xeon CPU and a single NVIDIA GeForce RTX 3090 GPU (24 GB VRAM). The model was optimized end-to-end without backbone freezing or token pruning. We employed the AdamW optimizer (betas = (0.9, 0.999), eps = 1 × 10^−8^) with a weight decay of 1 × 10^−5^ and a batch size of 8. The learning rate followed a linear warmup (starting at 1 × 10^−^loss (β^6^) followed by a cosine annealing schedule reaching a minimum of 1 × 10^−5^. The objective function combined a primary MSE regression loss with an auxiliary ranking = 0.5). Training was conducted for a maximum of 100 epochs, utilizing early stopping with a patience of 15 epochs based on the validation MAE. The model checkpoint achieving the lowest validation MAE was selected for final evaluation.

#### 2.3.1. CoTResNet3D

This study employs a ResNet-style 3D convolutional backbone, designed to capture the spatial continuity and anatomical heterogeneity inherent in voxelwise brain imaging data. Building upon this robust backbone we integrate two structural enhancement modules to improve the modeling of local dependencies and channel saliency: the CoT module and the Squeeze-and-Excitation 3D (SE3D) module. Specifically, the CoT module is designed to capture contextual relationships within 3D neighborhoods, and the SE3D module adaptively reweights channels after semantic refinement. These modules work synergistically to preserve convolutional priors and hierarchical scaling while endowing the network with greater structural sensitivity and robustness.

The CoT module is strategically inserted into each enabled residual block after the second 3D convolution and its batch normalization, but before SE3D module. This ordering ensures that conventional convolutions first extract local features, after which the CoT module refines spatial relationships using context-dependent dynamic kernel. Channel attention via SE3D is then applied to perform importance reweighting. By default, CoT is enabled only in stage two and stage three, while the first stage remains purely convolutional to preserve low-level texture encoding. This hierarchical design allows the network to retain stable encoding of fundamental features in the early layers while progressively refining the representation at higher levels. Conceptually, the CoT module generates context-dependent dynamic weights for each voxel location over a 3 × 3 × 3 neighborhood. In implementation, a grouped 3D convolution with kernel size 3 and four groups produces a context embedding. The embedding is concatenated with the original features along the channel dimension and passed through a 1 × 1 × 1 convolution to reduce to a hidden width equal to one eighth of the input channels, followed by another 1 × 1 × 1 convolution that outputs *K* = 27 kernel weights for the 3 × 3 × 3 neighborhood. We adopt a shared-weight scheme in which all channel groups reuse the same set of dynamic weights to balance stability and efficiency. To control computation, both the weight branch and the convolved branch are spatially downsampled by average pooling with a factor of 2 at each stage. The predicted weights are then upsampled by trilinear interpolation to the grid of the convolved branch, applied as a softmax over the 27 unfolded neighborhood positions, and aggregated by a weighted sum. A final 1 × 1 × 1 projection restores the original channel width, after which a learnable scalar gate, initialized to 0.5, modulates the contextual correction before it is added to the residual input.

**Figure 6:**
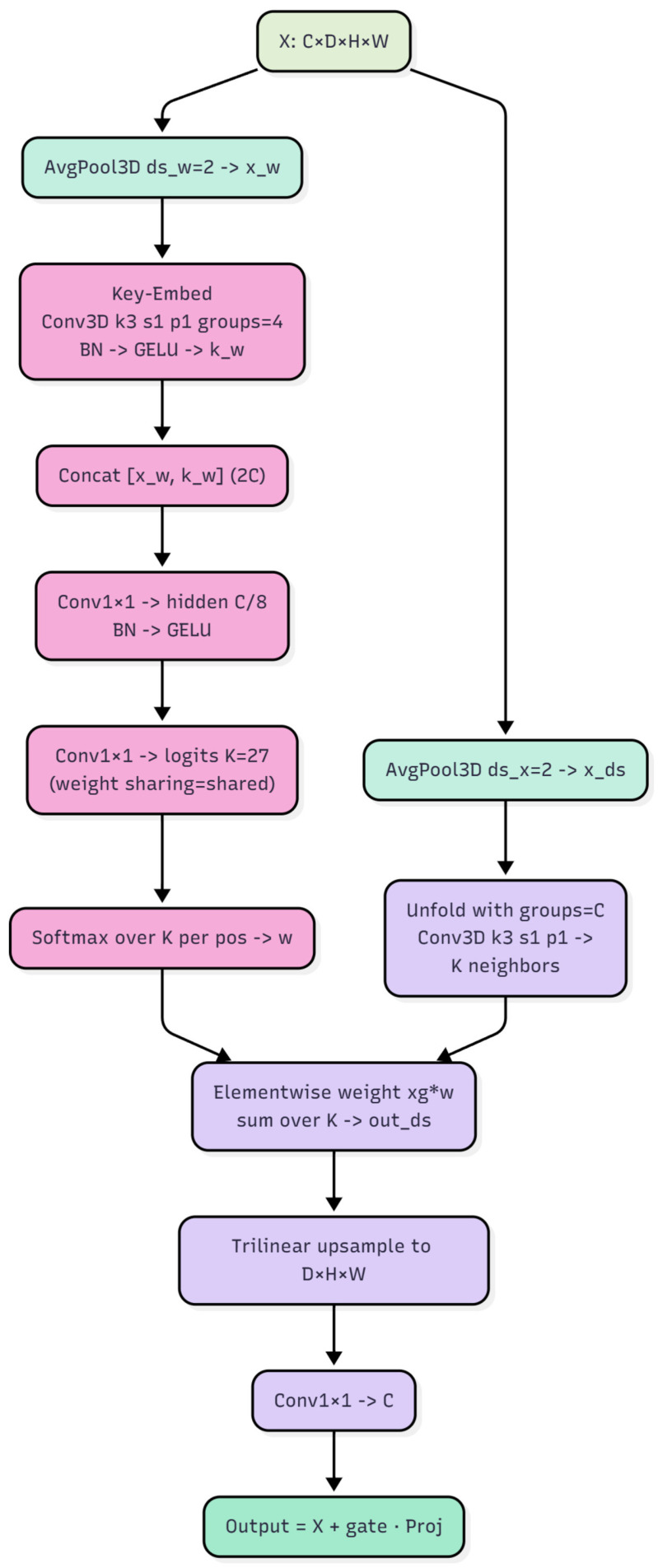
Detailed architecture of the CoT module.

**Figure 7:**
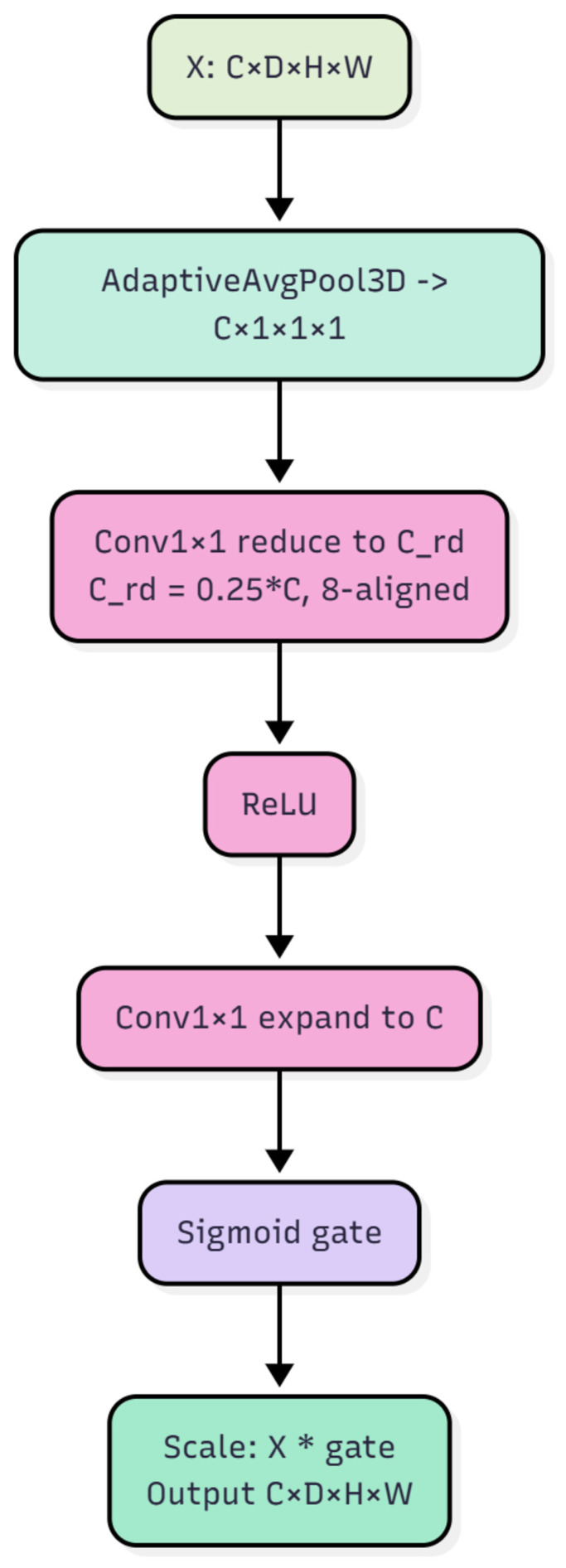
Detailed architecture of the SE3D module.

The SE3D module is placed in each residual block after the second convolution and batch normalization, and specifically after CoT but before 3D dropout. Its role is to perform channel-wise selective amplification and suppression once spatial relations have been refined by the dynamic kernels. Concretely, each channel is first summarized by 3D adaptive average pooling to a 1 × 1 × 1 global statistic, which is passed through two 1 × 1 × 1 convolutions with an intermediate ReLU to produce a channel-level sigmoid gate. The gate is then applied to the original features via channel-wise multiplication. The hidden width uses a reduction ratio of one quarter while being constrained to a multiple of eight, which preserves representational capacity, controls parameter count, and stabilizes training. The detailed architectures of the CoT and SE3D modules are illustrated in Figure 6 and Figure 7, respectively.

The proposed model operates on T1w MRI data with an input voxel size of 84 × 102 × 84 and a single channel. The backbone begins with a 7 × 7 × 7 3D convolution layer with stride 2 and symmetric padding, ensuring the preservation of boundary information. This is followed by batch normalization and a ReLU activation function, after which a 3 × 3 × 3 max pooling layer with stride 2 is applied. At this point, the spatial resolution is reduced to one-quarter of the original input size, yielding a feature map of 21 × 26 × 21 with 64 channels, establishing a stable low-level feature representation.

Stage 1 consists of two basic residual blocks, each containing two 3 × 3 × 3 convolutions, followed by batch normalization and ReLU activation. Both convolutions use stride 1, ensuring that spatial dimensions remain 21 × 26 × 21 with 64 channels. In this stage, the CoT module is not incorporated so the feature representation is built solely by convolutions, which effectively capture boundaries and textures. Stage 2 also contains two residual blocks. In the first block, the first 3 × 3 × 3 convolution in the main branch uses stride 2 to perform spatial downsampling, reducing the feature map size from 21 × 26 × 21 to 11 × 13 × 11, while increasing the number of channels to 128. In this stage, a CoT module is inserted after the second convolution and batch normalization within each block, enabling the model to capture 3D structural relations via dynamically adjusted neighborhood weights. This is followed by an SE3D module to emphasize age-relevant channel features and suppress redundant responses. Additionally, a 3D dropout layer with a drop rate of 0.1 is placed at the end of each block to mitigate overfitting.

**Figure 8:**
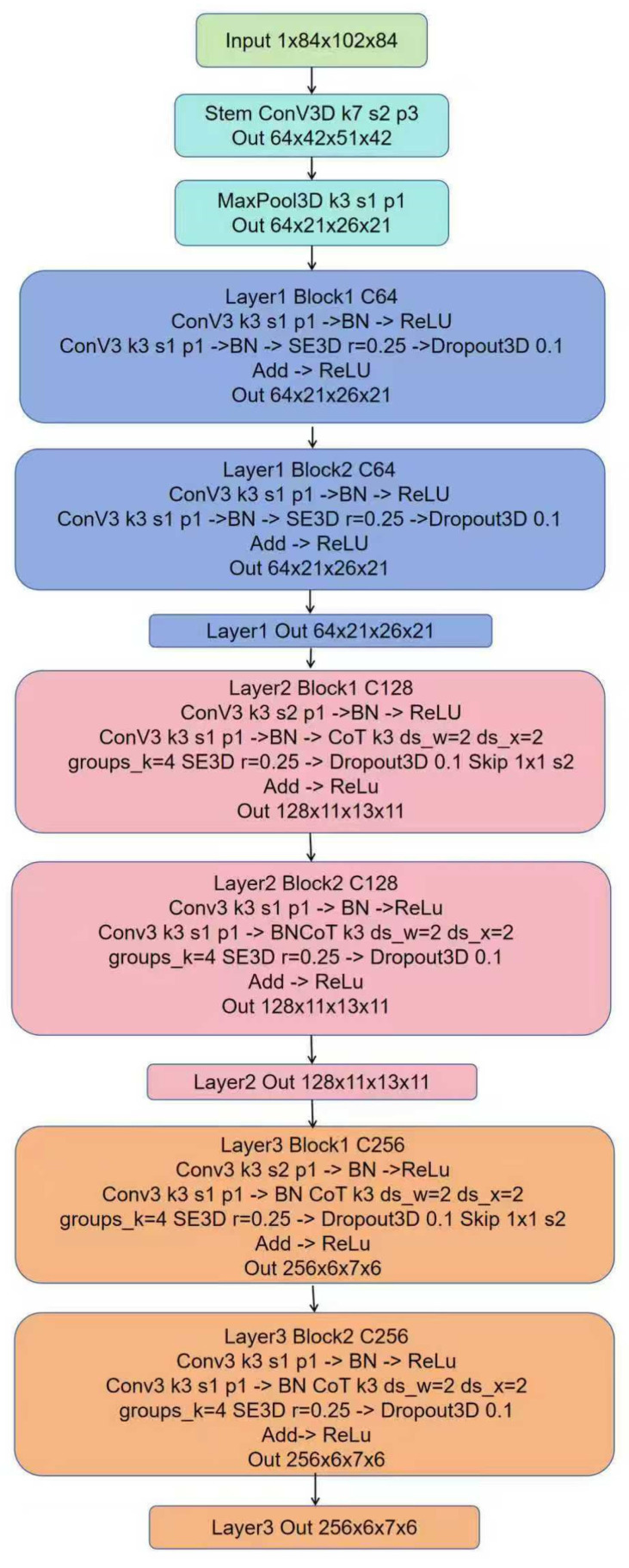
Detailed Hybrid ResNet-CoT architecture.

Stage 3 maintains the same internal structure as Stage 2. The first block again applies stride 2 to reduce feature map size from 11 × 13 × 11 to 6 × 7 × 6, while increasing the channels to 256. The expanded receptive field, combined with the stacked CoT and SE3D modules, enable the network to capture slowly varying macroscopic morphology together with cross-regional coordination patterns, which is directly beneficial for brain-age regression with a continuous target variable. The overall Hybrid ResNet-CoT architecture is illustrated in Figure 8.

#### 2.3.2. CrossViT

To further model voxel-level information at a global scale, we incorporate a CrossViT head on top of the 3D backbone, which receives two backbone feature maps to form small and large token streams. The large stream takes layer 2 features with 128 channels, while the small stream takes layer 3 features with 256 channels. Each stream is passed through a 3D convolutional PatchEmbed3D layer, which divides the voxel features into non-overlapping cubes and applies a linear projection using a 2 × 2 × 2 kernel and stride with no padding. The embedding dimensions are set to 96 for the small stream and 160 for the large stream. After embedding, features are reshaped from B × C × D × H × W to B × N × C, where N equals D × H × W after slicing. A learnable class token (cls) is then prepended to each sequence, with dimensions 1 × 1 × 96 for the small stream and 1 × 1 × 160 for the large stream. The parameters are initialized with truncated normal distribution, with a standard deviation 0.02. To preserve 3D geometry, each patch token receives a 3D sinusoidal positional encoding. The encoding is divided along the z, y, and x axes and concatenated along the last dimension. Concretely, we divide embedding dimensions by 3 to get a base value, adjust it to an even number, and distribute it across the axes. If the sum exceeds embedding dimension, the excess is subtracted from the x axis to maintain evenness, resulting in a 32 + 32 + 32 split for the 96-dimensional small stream and a 54 + 54 + 52 split for the 160-dimensional large stream. The positional encoding is added token-wise to patch embeddings but is not applied to the cls token. With positional and class tokens injected, each stream is refined by self-attention blocks to capture scale-specific context. Bidirectional cross-attention then enables interaction between the small and large streams, allowing for cross-scale global modeling and fusion.

After tokenization, each stream undergoes two layers of self-attention. A single self-attention layer consists of LayerNorm (LN) plus multi-head self-attention with a residual connection, followed by LN plus an MLP with a residual connection. The number of heads is fixed at 4, so the per-head dimension is 24 for the small stream and 40 for the large stream. The MLP expansion ratio is 4, which gives a hidden size of 384 for the small stream and 640 for the large stream. Two forms of stochastic regularization are used inside the block: the attention output dropout attn_drop at 0.1, and the MLP and generic block dropout at 0.1. We also adopt stochastic depth via DropPath with a total drop_path_rate of 0.1. Under depth equal to 2, the DropPath probabilities are 0.0 and 0.1 for the first and second layers respectively. Each residual branch is scaled by LayerScale with an initial gamma of 1 × 10^−4^, which helps numerical stability and gradient control in early training. Self-attention models long-range dependencies within a single scale and semantically complements the backbone convolutions that focus on local structures, thereby improving semantic consistency and global aggregation within each scale.

Cross-scale fusion is implemented by a CrossAttentionBlock with a bidirectional interaction mechanism. The small-stream cls is first linearly projected from 96 to 160 and used as the query to attend to the large-stream patches (which are LN beforehand). The cross-scale update is then linearly projected back to 96 and added residually to the small-stream cls. The reverse direction is symmetric: the large-stream cls is projected from 160 down to 96 to query the small-stream patches, then the update is projected back to 160 and added residually to the large-stream cls. Each direction has its own LN and MLP, and inherits the same DropPath and LayerScale settings as in self-attention. To further control the strength of cross-scale information injection, the cross updates are multiplied by learnable scalar gates, gate_s for the small stream and gate_l for the large stream, both initialized to 0.1. The CrossViT head is stacked to a depth of two layers, and each layer executes in the order of small self-attention, large self-attention, and bidirectional cross-attention. This progressively reinforces the complementarity between fine-grained detail and macroscopic context, allowing the small stream to focus on anatomical structure while the large stream supplies stable global context, and together they improve global discriminability.

For token-level training, two practical mechanisms are introduced to balance efficiency and generalization. First, a token retention rate token_keep_rate controls stochastic token pruning during training: the default 1.0 keeps all tokens; values below 1.0 randomly retain only a subset of patch tokens, which reduces attention cost and acts as sparsity regularization to improve robustness. Second, an optional backbone-freezing strategy freeze_backbone is provided for limited-sample medical imaging: initially freeze the convolutional backbone and train only the Transformer blocks and the regression head; after convergence, unfreeze the backbone for joint fine-tuning. This mitigates early overfitting and improves training stability.

**Figure 9:**
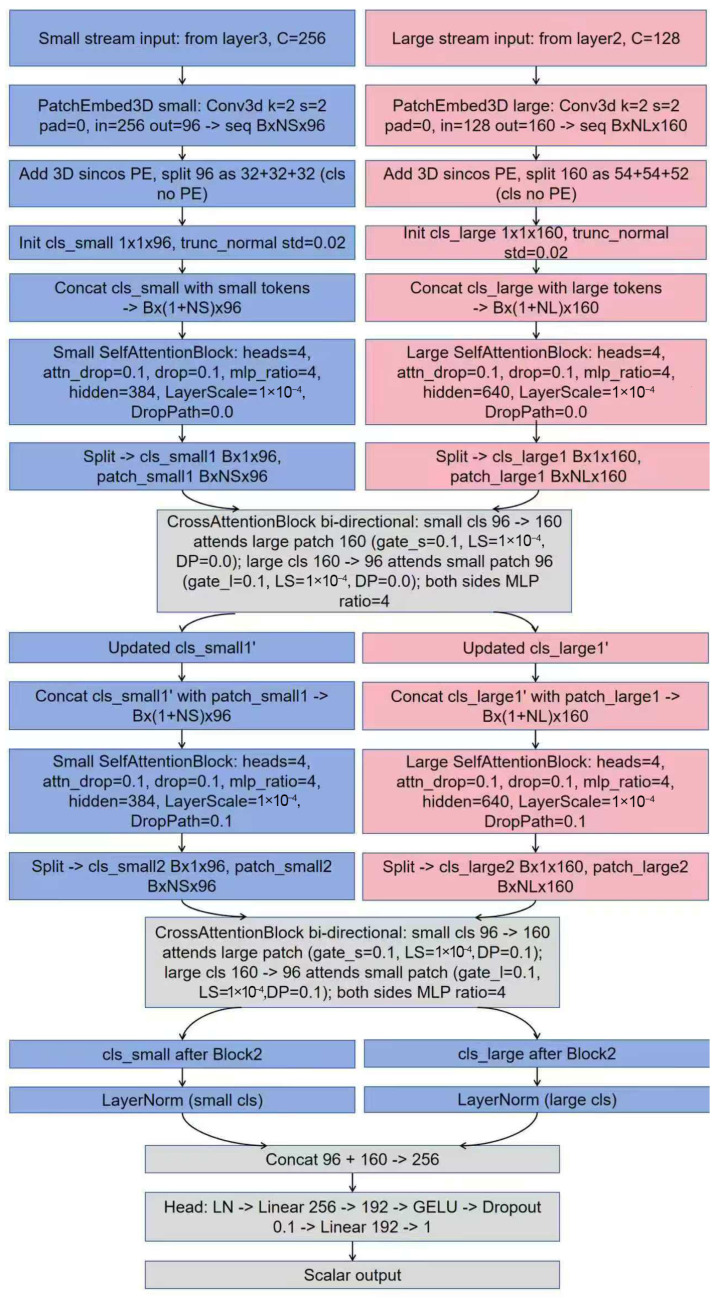
Detailed CrossViT architecture.

**Figure 10:**
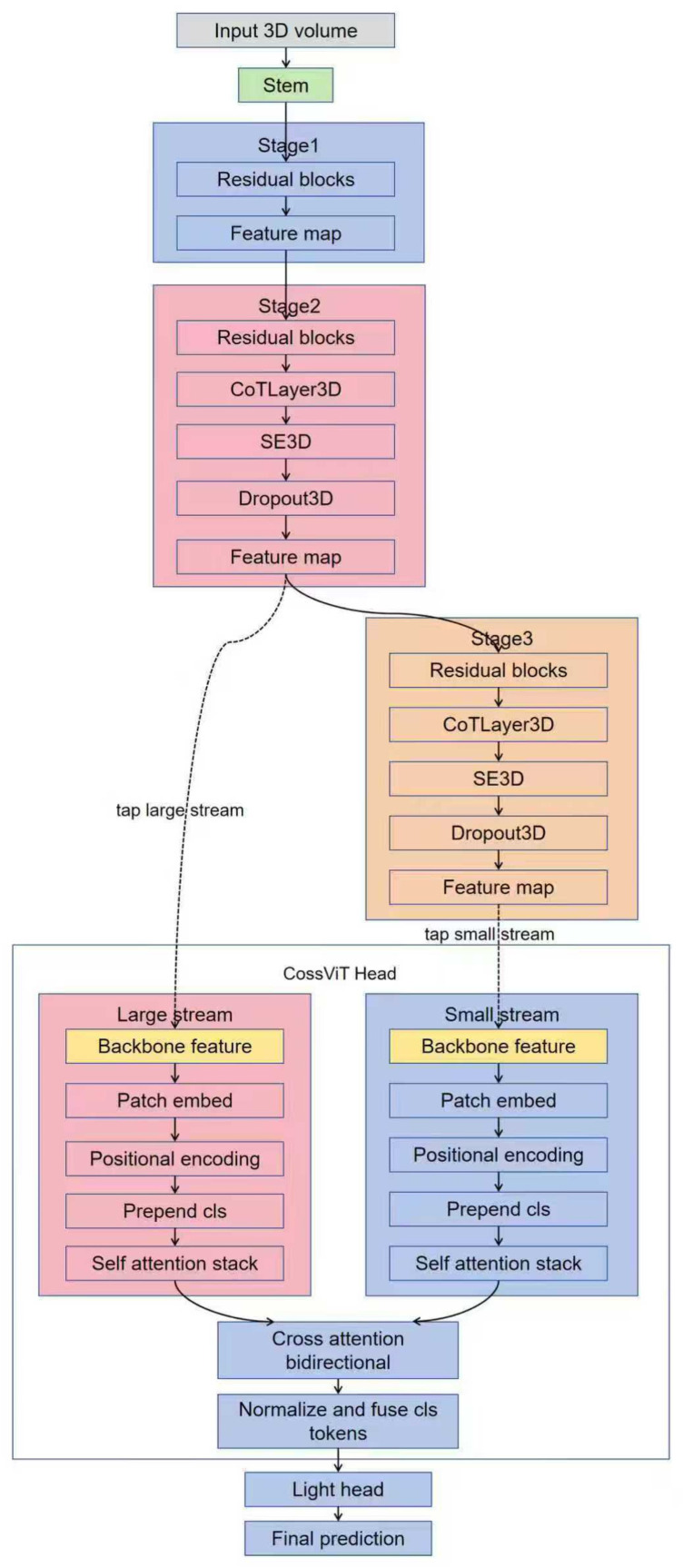
Overall model architecture.

After fusion, the model takes the cls tokens from the small and large streams, applies a per-stream LN, and concatenates them along the channel dimension to form a 256-dim vector, which is fed to a lightweight regression head. The head consists of LN, a linear layer mapping 256 to 192, GELU activation, Dropout of 0.1, and a final linear layer that outputs a 1-D scalar suitable for regression tasks such as individual brain-age prediction. This design keeps cross-scale interactions rich while remaining compact and efficient, with clear hyperparameters: patch size and stride 2 × 2 × 2, embedding dimensions 96 (small) and 160 (large), 4 attention heads, MLP expansion ratio 4, attn_drop 0.1, dropout 0.1, DropPath probabilities 0.0 and 0.1 for the two layers, LayerScale initialized to 1 × 10^−4^, and cross-attention gates initialized to 0.1. Through this pipeline, 3D voxel features are reliably converted into cross-scale semantic tokens and aligned and enhanced from global to local by the joint effect of self- and cross-attention. Figure 9 and Figure 10 depict the CrossViT head and the overall fusion model, respectively.

## 3. Results

To systematically evaluate the contribution of each individual component within our architecture, we conducted a comprehensive ablation study. The proposed ResNet-CrossViT was compared against three ablated B models representing different structural configurations.

ResNet-only B: This model adopts a three-stage network architecture identical to the CNN branch of our full model. By isolating the convolutional stream, we can assess the performance of local feature extraction in isolation while ensuring architectural consistency for a fair comparison.

ViT-only B: This model employs a patch size of 16 × 16 × 16, resulting in 150 patches and a cls token. By feeding these tokens into a standalone 12-layer Transformer encoder, this configuration evaluates the model’s capacity to estimate brain age using only global self-attention mechanisms.

ResNet-ViT fusion B: This model integrates the aforementioned CNN and ViT streams using a straightforward feature concatenation strategy. Crucially, this baseline lacks the Cross-Attention mechanism, serving to quantify the specific performance gains attributable to our proposed inter-branch information exchange.

To ensure a fair comparison, all models are trained and evaluated under identical experimental settings, using the same data preprocessing pipeline and data partitioning scheme.

### 3.1. Performance Evaluation on the Internal Dataset

Table 2 summarizes the comparative performance of the proposed ResNet-CrossViT and the three B models on the complete internal test set. To further examine model performance across the human lifespan, Table 3, Table 4 and Table 5 detail the results across three biologically distinct age cohorts: [0, 18), [18, 60), and [60, 96) years. This partitioning is strategically chosen to represent the critical stages of brain maturation, stable adulthood, and senescence, respectively. Specifically, the [0, 18) group captures the period of rapid neurodevelopment and pruning, the [18, 60) group represents the plateau of structural maturity, and the [60, 96) group encompasses the phase of age-related neurodegeneration and structural atrophy. By evaluating these stages separately, we can better assess the model’s sensitivity to the diverse morphological changes characteristic of different life phases.

Specifically, the MAE, which directly quantifies point-wise prediction accuracy, is reduced to 2.72 years for ResNet-CrossViT, a substantial improvement over ResNet (4.49 years), ViT (4.58 years), and ResNet-ViT (3.52 years). This significant reduction indicates that the proposed model achieves more precise individual-level brain age estimation. Meanwhile, the ME of ResNet-CrossViT is merely 0.05 years, suggesting near zero systematic bias and implying that the residual errors are primarily attributable to random fluctuations rather than directional offsets. In contrast, the ResNet and ResNet-ViT exhibit varying degrees of overall overestimation or underestimation. Furthermore, the high *r* and *R*^2^ attained by ResNet-CrossViT confirm its exceptional capacity to capture age related trends. Notably, ResNet-CrossViT also achieves the lowest mMAE (5.10), representing a more than two-fold improvement over ResNet (10.98) and a substantial reduction compared to ResNet-ViT (10.40). These findings collectively demonstrate that ResNet-CrossViT effectively reduces overall prediction error while maintaining a balanced error distribution across specific age intervals, thereby mitigating extreme mismatches.

**Figure 11:**
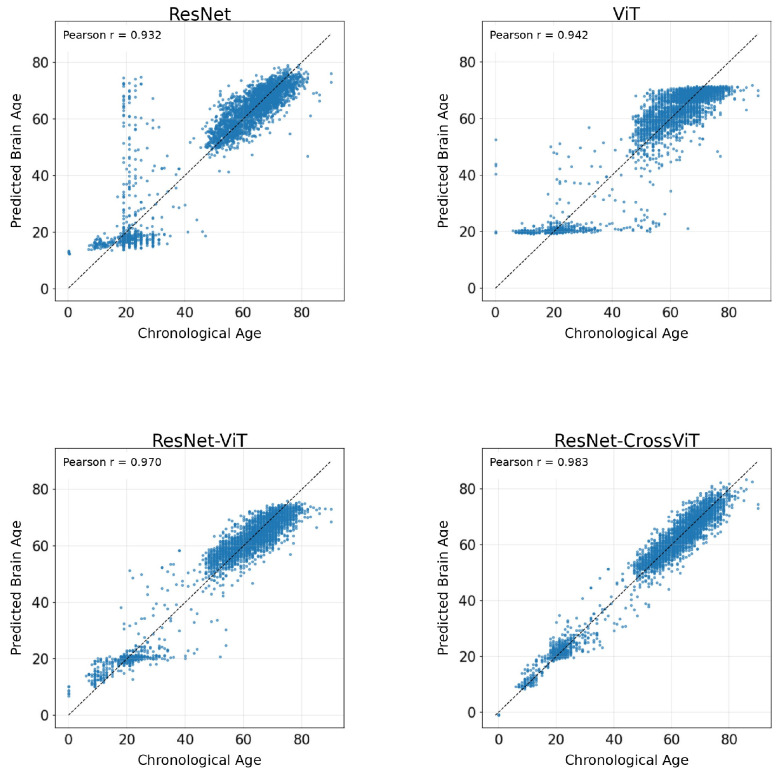
Scatter plots of predicted brain age versus chronological age for different models on the test set ((**Top left**): ResNet; (**top right**): ViT; (**bottom left**): ResNet-ViT; (**bottom right**): ResNet-CrossViT). The dot line represents the identity line, where a perfect match between predicted and chronological age would occur.

The visual evidence in Figure 11 corroborates the quantitative superiorities reported in Table 2, offering a more intuitive perspective on the architectural advantages of ResNet-CrossViT. While the B models (ResNet and ViT) demonstrate a general positive correlation, they exhibit significant dispersion and prominent prediction “plateaus”—horizontal clusters where the models fail to differentiate age-specific morphological nuances. Although the ResNet-ViT fusion baseline significantly tightens the distribution, it still maintains a noticeable “cloud” of residual dispersion among individuals between the ages of 20 and 50, suggesting that simple feature concatenation lacks the representational depth required to fully resolve the subtle morphological variations and non-linear structural transitions occurring during this period of stable adulthood. In contrast, ResNet-CrossViT achieves the most precise alignment with the identity line, yielding a highly linearized diagonal band that remains consistent across the entire lifespan. This lack of systematic “plateauing” and the reduced dispersion in both young and elderly subjects confirm that the cross-scale interaction mechanism effectively rectifies regional prediction biases. By integrating local structural details with global context, the proposed model ensures that both rapid neurodevelopmental milestones and progressive neurodegenerative trends are mapped with exceptional fidelity.

In the age-stratified evaluation (Table 3, Table 4 and Table 5), the child–adolescent group ([0,18) years) presented the most formidable challenge for brain-age prediction. As shown in Table 3, B models (ResNet, ViT, ResNet-ViT) exhibited significantly elevated MAE and positive ME. Notably, the occurrence of negative *R*^2^ values—most notably for the ViT model (*R*^2^ = −9.38) in the pediatric cohort—signifies a total breakdown of predictive capacity within this age range. Statistically, a negative *R*^2^ indicates that the model’s residual sum of squares exceeds the total sum of squares around the mean, implying that the model performs worse than a horizontal line representing the group’s average age. This performance degradation is not merely a sampling artifact but stems from the profound biological complexity of this period [36,37], characterized by highly nonlinear maturation trajectories and marked regional asynchrony in myelination and gray-matter remodeling [38,39,40]. In contrast, ResNet-CrossViT effectively mitigated these difficulties, maintaining a robust *R*^2^ (0.54) and the lowest MAE (1.95 years) among all tested architectures.

For the adult cohort ([18, 60) years), which represents a plateau of structural maturity, all models showed improved stability. However, ResNet-CrossViT remained the superior performer, reaching an exceptional Pearson correlation (*r* = 0.98) and *R*^2^ (0.95), suggesting that its cross-scale interaction mechanism captures subtle aging cues that simpler architectures overlook during this stable phase.

In the elderly and senescent cohort ([60, 97) years), a distinct shift in error patterns was observed. Unlike the overestimation seen in the younger group, models here tended toward systematic underestimation (ME < 0), likely reflecting the accelerated and heterogeneous nature of late-life brain atrophy. ResNet-CrossViT minimized this bias (ME = −0.66) compared to ResNet-ViT (ME = −1.96), confirming that the integration of global context with local details provides a more faithful mapping of progressive neurodegeneration.

Overall, the consistent superiority of ResNet-CrossViT across these biologically distinct stages—from the high-variance neurodevelopment of youth to the subtle transitions of adulthood and the atrophy of old age—highlights its exceptional robustness and potential for lifespan-wide clinical application.

### 3.2. Performance Evaluation on the Unseen Dataset

Table 6 presents the evaluation metrics on the unseen dataset, providing a stringent assessment of model generalization across domain shifts involving different acquisition protocols and demographic distributions. Under these challenging conditions, ResNet-CrossViT demonstrates a superior performance envelope. Specifically, it achieves an MAE of 4.19 years, representing a substantial error reduction compared to ResNet (7.15 years), ViT (7.98 years), and ResNet-ViT (6.37 years). In addition, ResNet-CrossViT attains the lowest mMAE (6.40), suggesting its exceptional ability to suppress worst-case prediction errors across age intervals even when subjected to significant distributional variations.

Regarding systematic bias, while ResNet and ViT exhibit pronounced underestimation (ME of −3.15 and −2.69 years, respectively), ResNet-CrossViT maintains a near-zero ME (−0.28 years), effectively alleviating the directional shifts typically induced by cross-domain transfer.

**Figure 12:**
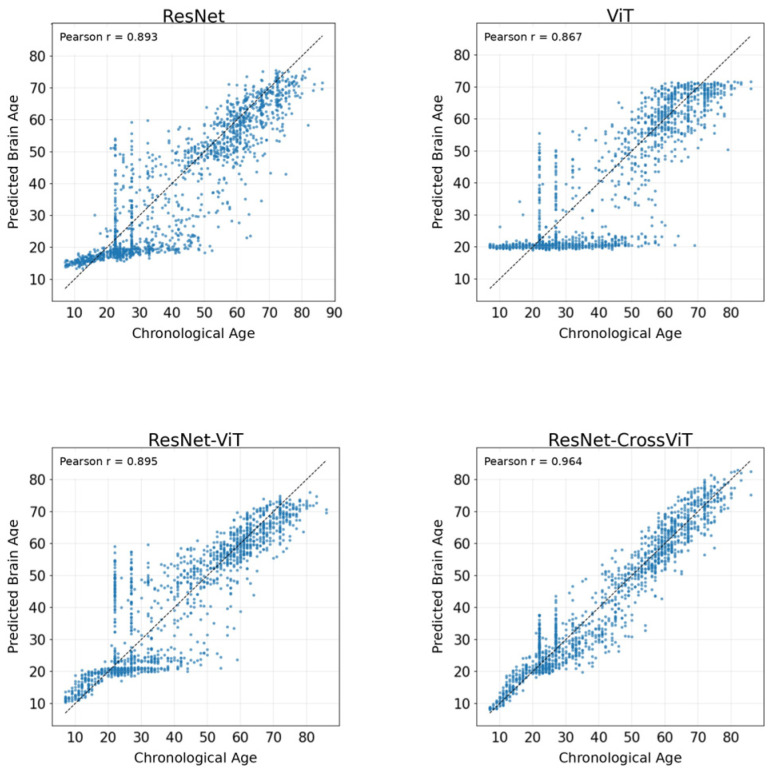
Scatter plots illustrating the correlation between predicted brain age and chronological age for various models on the unseen dataset. ((**Top left**): ResNet; (**top right**): ViT; (**bottom left**): ResNet-ViT; (**bottom right**): ResNet-CrossViT).

The scatter plots in Figure 12 further corroborate these findings. Despite the increased dispersion inherent in unseen data, ResNet-CrossViT yields a Pearson correlation of 0.964, markedly outperforming ResNet (0.893), ViT (0.867), and ResNet-ViT (0.895). The tighter clustering of data points around the identity line (Y = X) confirms that the cross-scale interaction mechanism preserves age-related structural trends with high fidelity, even under domain-shifted conditions. Collectively, these results validate the robust generalization and architectural stability of ResNet-CrossViT for lifespan brain-age estimation in diverse clinical settings.

### 3.3. Consistency Evaluation on the Longitudinal Dataset

To further evaluate the models’ ability to capture individual-level brain aging trajectories, we assessed their predictive consistency on the longitudinal dataset, with the results summarized in Table 7. Unlike cross-sectional analysis, longitudinal validation focuses on the temporal evolution of structural cues within the same individual, providing a more rigorous test of a model’s sensitivity to subtle, age-related progression.

As shown in Table 7, ResNet-CrossViT achieves superior performance across all change-related metrics, yielding an MdE = −0.50, an MAdE = 3.68, and an mMAdE = 6.02. These results represent a significant error reduction compared to both the ResNet-ViT B (MAdE = 4.91) and the standalone ViT architecture, which exhibited substantial instability in follow-up settings (MAdE = 8.86). Notably, the MdE for ResNet-CrossViT (−0.50) is the closest to zero among all tested models, indicating that it effectively minimizes overall deviation in estimated changes. The marked reduction in mMAdE (6.02) is noteworthy; it suggests that ResNet-CrossViT effectively constrains worst-case prediction errors, maintaining higher reliability even for individuals with atypical aging patterns. These findings suggest that the cross-scale interaction mechanism is more adept at resolving fine-grained structural plasticity than direct feature concatenation, enabling the model to track individual brain aging with higher temporal fidelity and robustness.

### 3.4. Reproducibility Evaluation on the Retest Dataset

In addition to longitudinal consistency, we further evaluate model reproducibility under repeated scanning conditions through test–retest analysis on a dedicated dataset, with the results detailed in Table 8. The retest dataset primarily reflects the stability of model outputs in the presence of realistic acquisition noise and scan-related variability, and thus serves as an important indicator of reliability for brain age tracking.

As shown in Table 8, ResNet-CrossViT achieves the highest ICC (0.994) among all models, approaching the level of near-perfect agreement. This indicates superior overall consistency and reliable subject ranking across repeated measurements. Moreover, ResNet-CrossViT attains the lowest standard deviation of differences (*σ*(*d*) = 1.71), demonstrating the smallest variability in prediction differences between repeated scans and thus the best difference stability. Although its scan-wise prediction standard deviation (*σ*(*y′_scan*) = 0.56) is not the lowest, ResNet-CrossViT remains optimal on the more critical reproducibility metrics, namely ICC and *σ*(*d*). These findings suggest that the proposed architecture is highly resilient to acquisition noise and technical variability, resulting in superior test–retest reliability.

## 4. Discussion

This study addresses the central question of how to achieve clinically meaningful brain-age estimation across the human lifespan, not only low pointwise error, but also stable and temporally reliable estimates under realistic multi-site variability and repeated scanning. To this end, we constructed a large-scale, multicenter T1-weighted MRI dataset spanning ages 0 to 96 years and proposed a hybrid ResNet-CrossViT architecture that integrates the complementary strengths of convolutional neural networks and Transformers. Through systematic evaluation on internal test data, unseen datasets, longitudinal datasets, and test–retest datasets, we validated the effectiveness of the proposed model from multiple perspectives, including predictive accuracy, age-stratified robustness, longitudinal consistency, and reproducibility. In the following, we interpret these findings in the context of prior studies and provide an in-depth discussion of model performance advantages, age-dependent differences, cross-domain generalization capability, as well as potential limitations and future research directions.

### 4.1. Overall Advantages of ResNet-CrossViT in Lifespan Brain-Age Prediction

From a methodological perspective, the central advantage of ResNet-CrossViT lies not merely in improved pointwise accuracy, but in its ability to provide stable, unbiased, and lifespan-consistent brain-age estimates across heterogeneous developmental and aging stages. In lifespan brain-age prediction, while local accuracy provides a necessary foundation, the key challenge lies in maintaining prediction stability, trend consistency, and statistical interpretability across fundamentally of different phases of neurodevelopment and neurodegeneration. Human brain structure follows markedly different trajectories from infancy to older age, and models based on a single architectural paradigm often struggle to accommodate both rapid, locally driven developmental changes and globally coordinated degenerative patterns, resulting in substantial variability in lifespan-wide prediction.

During infancy and adolescence, brain development involves rapid, nonlinear, and highly heterogeneous local structural changes, including cortical maturation, myelination, and gray-matter remodeling. Effective modeling at this stage requires strong sensitivity to fine-grained local morphological features; otherwise, genuine developmental variability may be misinterpreted as noise, undermining prediction stability. In adulthood, structural changes become subtle and spatially diffuse, increasing the risk of prediction compression or “plateau effects” in models lacking sufficient sensitivity. In older age, brain aging is characterized by coordinated changes across distributed degeneration across distributed networks rather than isolated regional atrophy, placing greater demands on the modeling of long-range dependencies and global structural associations.

ResNet-CrossViT is well aligned with these stage-specific neurobiological characteristics through its hierarchical architectural design. The convolutional backbone preserves inductive biases toward local anatomical structures, supporting robust characterization of rapidly evolving morphology during development. The integration of context-aware dynamic feature further enhances sensitivity to neighborhood-level structural relationships, improving robustness in highly heterogeneous populations. At higher feature levels, cross-scale Transformer interactions enable effective integration of information across brain regions and spatial scales, facilitating the extraction of coherent aging trends from dispersed and weak signals. This hierarchical fusion reduces reliance on isolated regional cues, suppresses systematic bias, and preserves consistency in age-related trajectories across the lifespan.

ResNet-CrossViT demonstrates competitive performance relative to several contemporary Transformer and Transformer-hybrid brain-age architectures, achieving an MAE of 2.72 years while maintaining end-to-end 3D T1-weighted volumetric processing (Table 9). While direct performance comparisons in Table 9 are naturally subject to variations in cohort composition and preprocessing across studies, our model’s ability to preserve full spatial context—in contrast to approaches relying on 2D slices or ROI features—likely contributes to its robust cross-center generalization. This hybrid architecture presents potential for enhancing clinical viability in lifespan brain age estimation and establishes a notable benchmark among Transformer-driven solutions.

### 4.2. Hierarchical Hybrid Modeling Improves Brain-Age Prediction Across Youth and Adolescence

Childhood and adolescence represent an intrinsic instability regime for lifespan brain-age modeling, driven jointly by neurobiological nonlinearity and statistical learning constraints. Brain age modeling across the entire lifespan reveals significant performance disparities across different age cohorts, and while predicting age during childhood and adolescence may seem straightforward in localized models due to abundant structural signals, it remains a formidable challenge within a comprehensive lifespan framework. Age-stratified analyses confirm that, although local models can achieve accurate predictions in this interval, integrating these ages into full-lifespan models introduces instability. Importantly, this pattern is not unique to the present study, but rather a recurring observation reported across multiple lifespan brain-age investigations [44,45].

From a neurobiological perspective, the predictive instability during childhood and adolescence arises not merely from the intrinsic complexity of development, but from the profound structural divergence of these early-life signatures compared to the rest of the lifespan. While neurodevelopmental processes—such as cortical thinning, white matter myelination, and gray matter remodeling—are highly dynamic, they exhibit age-specific morphological motifs that are qualitatively distinct from the patterns of adulthood and senescence. These early signals are characterized by regional asynchrony and intense nonlinearity, creating a sharp contrast with the more spatially diffuse and linear atrophy trends observed in later life. In a unified lifespan model, this structural asymmetry poses a significant challenge: the model must reconcile the high-frequency, heterogeneous signals of rapid growth with the dominant, large-scale signals of aging. Consequently, the unique neurobiological signatures of youth are often poorly generalized or overshadowed within a global framework, leading to increased prediction errors and systematic bias.

From a statistical learning perspective, within a lifespan regression framework, gradient descent trajectories are typically steered by the empirical risk associated with denser, more homoscedastic age ranges—namely adulthood and older age—effectively marginalizing the unique feature-age mappings of the developmental cohort during training. This phenomenon, compounded by the heteroscedasticity of early-life brain structures and sample-size imbalances, forces the global optimizer to prioritize the majority distribution, leading to a representational mismatch on the lower-density developmental manifold [46,47]. Our experimental results confirm this failure to capture high-frequency developmental variances: standalone ResNet, ViT, and ResNet–ViT architectures consistently struggle with out-of-distribution generalization in the early life stage, exhibiting substantially larger MAE, pronounced positive systematic bias (ME), and collapsing correlations—where a negative *R*^2^ indicates the models perform worse than a horizontal mean baseline within the [0, 18) interval.

In contrast, ResNet-CrossViT substantially improves brain-age prediction performance in the [0, 18) group, as evidenced by reduced MAE and mMAE alongside positive and relatively high correlations. This suggests that the proposed hybrid architecture can partially alleviate the modeling difficulty arising from the combination of adolescence-specific structural heterogeneity, as well as nonlinear and asynchronous neurodevelopmental trajectories. Mechanistically, the CoT component strengthens local contextual modeling within the convolutional stream, enabling the network to capture fine-grained and rapidly changing developmental details that are often highly nonlinear at the regional level. Meanwhile, the CrossViT module performs cross-scale token interactions to fuse multi-resolution representations and to integrate global maturational trends, effectively reconciling the structural divergence between youth and adult/older brain features. This hierarchical combination preserves sensitivity to local neurodevelopmental variations while maintaining a robust mapping to chronological age, thereby improving the stability and reliability of brain-age estimates across childhood and adolescence.

### 4.3. Cross-Center Generalization: Clinical Translation and Mechanistic Insights

Cross-center generalization remains one of the most critical and unresolved challenges for the clinical translation of brain-age models. For brain-age estimation to be viable in real clinical settings, the primary hurdle is not merely achieving a slightly lower MAE on an in-distribution test set, but rather maintaining acceptable prediction error and stable systematic bias across different sites, scanners, and acquisition protocols. MRI data are inevitably affected by variations in scanner vendors, field strengths, sequence parameters, and population characteristics. If a model relies excessively on site-specific “imaging style” features, it is prone to systematic drift under domain shift, which can substantially undermine clinical usability.

In this context, the unseen dataset used in the present study can be viewed as a practical proxy for real-world multi-center deployment. Because it differs markedly from the internal data in scanner hardware, acquisition protocols, and sample sources, it provides a more stringent test of whether the model truly learns age-related anatomical regularities rather than fitting idiosyncrasies of a specific training distribution. Prior work has consistently reported that many brain-age models perform well in-distribution but exhibit pronounced performance degradation on external datasets [48]. Such external evaluations often amplify sensitivity to domain shift and expose potential reliance on spurious correlations.

Under this challenging, deployment-like setting, our results provide direct evidence for the cross-center robustness of ResNet-CrossViT. On the unseen dataset, it achieved the lowest MAE (4.19 years) and mMAE (6.40 years), with systematic bias close to zero, clearly outperforming ResNet, ViT, and ResNet–ViT. Notably, in cross-center generalization, improved accuracy is not the only criterion—near-zero bias indicates the absence of a global drift after domain transfer, which is particularly important for clinical interpretation, cross-sectional comparability, and longitudinal follow-up.

Mechanistically, the robust cross-center generalization of ResNet-CrossViT may arise from its hierarchical ability to disentangle intrinsic biological signals from extrinsic technical noise. This is achieved through a synergy between our data strategy and architectural inductive bias. First, by intentionally preserving imaging heterogeneity during training, we force the model to prioritize broadly generalizable anatomical representations over site-specific imaging styles. At the architectural level, the CoT modules enhance local “structural-detail reliability” through context-aware dynamic modeling, encouraging the extraction of fine-grained morphological features that remain stable across protocols. Simultaneously, the CrossViT component acts as a structural regularizer; by leveraging bidirectional cross-attention, it integrates these local cues into a global representation of trend consistency. This global dependency modeling is particularly effective at suppressing site-specific artifacts—which are often spatially incoherent—while prioritizing coordinated, network-level maturation and aging patterns conserved across different scanners. Consequently, this hierarchical fusion ensures that the resulting brain-age trajectories are not only accurate but also biologically grounded and reproducible, providing a viable path for large-scale, real-world clinical applications.

### 4.4. Assessment of Test–Retest Reliability and Temporal Stability

For brain age to function as a clinically meaningful biomarker, stability across time and repeated measurements is often more critical than minimal pointwise error at a single time point, but in its ability to reliably track disease progression or therapeutic response. A model characterized by low mean error yet high temporal jitter—stochastic fluctuations in predicted age across short intervals—is insufficient for longitudinal monitoring. These properties are essential for real-world applications such as monitoring disease progression, evaluating intervention effects, and supporting individualized risk stratification based on a stable biological baseline.

Motivated by this translational requirement, the present study extends beyond static accuracy and systematically evaluates longitudinal consistency and test–retest reproducibility—two aspects that have received comparatively limited attention in many prior brain-age studies. Our results show that ResNet-CrossViT achieves the lowest MAdE and mMAdE on longitudinal data and exhibits near-perfect test–retest reproducibility (ICC = 0.994). These metrics imply that the model remains sensitive to temporal progression while maintaining stable subject discriminability even in the presence of non-biological variance, such as subtle differences in head positioning or hardware fluctuations that are often misinterpreted as “aging signals”.

Mechanistically, the superiority of ResNet-CrossViT stems from a hierarchical defense mechanism that disentangles genuine age-related structural changes from transient acquisition noise. On the one hand, CoT modules provide local structural anchoring by strengthening contextual relations, encouraging the model to rely on reproducible anatomical configurations rather than protocol-sensitive artifacts. On the other hand, the CrossViT component acts as a structural regularizer, constraining local evidence within a global trend through cross-scale fusion. By leveraging bidirectional cross-attention to achieve a “global consensus” of the distributed structural state, any transient local fluctuation in a single scan is effectively mitigated, thereby ensuring “trend consistency” across multiple time points.

Therefore, longitudinal consistency and test–retest reliability should be viewed not as ancillary metrics, but as key criteria determining whether brain age can progress from a research outcome to a clinically deployable tool. The advantages of ResNet-CrossViT on both evaluations suggest its potential utility in practical clinical tasks, including robust tracking of intra-individual change across repeated scans, assessment of treatment response, and individualized risk assessment and management.

### 4.5. Limitations and Future Directions

Despite the encouraging results obtained in this study, several limitations should be acknowledged. First, although our dataset spans the full lifespan (0–96 years), the age distribution remains inevitably imbalanced. In particular, samples at the extreme ends of the age spectrum—such as infants and the oldest-old—are still relatively sparse in certain intervals. This long-tail sparsity may reduce the statistical stability of age-stratified metrics within these ranges and can further limit the model’s ability to learn sufficiently robust mappings for rare age cohorts in a unified lifespan framework.

Second, the current model is trained exclusively on T1-weighted structural MRI. While T1w imaging is the most consistently available modality across large-scale public datasets and thus enables reliable multi-site aggregation, relying solely on structural information may restrict the model’s sensitivity to functional alterations, microstructural white-matter changes, and network-level reorganization that accompany aging and neuropsychiatric disorders. Future work could explore multimodal extensions by integrating diffusion MRI or fMRI into a unified architecture, potentially improving the characterization of complex aging mechanisms and enhancing sensitivity to pathological deviations from normative aging trajectories.

Third, although Transformer-based or Transformer-hybrid architectures provide strong capacity for modeling long-range dependencies and cross-scale interactions, they often introduce higher computational overhead and memory cost during inference, especially when processing volumetric 3D inputs [49]. This may impose practical constraints on deployment in resource-limited settings or time-sensitive clinical workflows. Therefore, future studies should investigate efficiency-oriented improvements, such as lightweight architectural design, sparse attention mechanisms, token pruning strategies, or knowledge distillation, to reduce inference burden while preserving predictive performance and stability.

Fourth, while our model was trained on a large-scale multi-site dataset to enhance generalizability, several source-level factors may still influence prediction precision. Inter-subject biological variability, such as atypical brain morphology not fully captured by the training distribution, remains an inherent challenge. Furthermore, despite our efforts to harmonize data, domain shift stemming from differences in scanner hardware and acquisition protocols can introduce non-biological variance. Residual sensor noise, including subtle motion artifacts, may also affect the stability of high-resolution feature extraction. Future work should integrate more advanced domain adaptation and automated quality control to further mitigate these environmental influences.

Fifth, while the use of standardized metrics and overlapping data sources in the recent literature provides a reasonable basis for performance benchmarking, a direct head-to-head comparison with all contemporary state-of-the-art (SOTA) models within a unified computational environment was constrained by the limited availability of open-source code and the prohibitive computational demands of training 3D-heavy architectures. Establishing a more direct and transparent benchmark across diverse model structures remains a primary objective of our future research.

In addition, although we evaluated robustness using multiple stability-related metrics (including cross-center generalization, longitudinal consistency, and test–retest reproducibility), the biological interpretability of the representations learned by the model warrants deeper investigation. Specifically, it remains important to systematically examine which neuroanatomical regions are most emphasized by the model, how their contributions vary across the lifespan, and whether these learned patterns align with established neurodevelopmental and neurodegenerative trajectories.

Future research may proceed in several directions. (1) Developing unified frameworks that integrate prediction and interpretability may further strengthen the neurobiological plausibility and clinical credibility of brain-age estimates. This could be achieved by incorporating explainable AI techniques—such as saliency-based analyses, attention visualization, or region-level contribution profiling—directly into the modeling pipeline to provide structured and reproducible explanations of the decision process. (2) Extending ResNet-CrossViT to specific patient populations is critical for evaluating its translational sensitivity and clinical utility. While our current experiments focus on healthy cohorts, brain-age biomarkers are most valuable when applied to clinical groups, where the goal is to detect accelerated aging, quantify disease-related deviation, and monitor progression over time. Future studies could apply the proposed model to cohorts with Alzheimer’s disease [50], Parkinson’s disease, or other neurological and psychiatric disorders [51], and assess whether the model can robustly track disease progression, capture treatment effects, and support individualized risk stratification and management.

Overall, while ResNet-CrossViT demonstrates strong accuracy, stability, and generalization potential for lifespan brain-age prediction, continued improvements in data coverage, multimodal integration, computational efficiency, and interpretability will be essential to facilitate broader clinical translation.

## 5. Conclusions

This study aimed to improve the stability, generalizability, and reliability of brain-age prediction across the human lifespan. We curated a large-scale multicenter T1-weighted MRI dataset spanning ages 0–96 years and proposed ResNet-CrossViT, a hybrid CNN–Transformer framework that integrates CoT-enhanced 3D convolutional feature extraction with cross-scale Transformer interactions. Across internal evaluation and multiple external validation settings—including unseen-center data, longitudinal follow-up data, and test–retest scans—ResNet-CrossViT consistently demonstrated competitive accuracy, reduced worst-case age-stratified errors, near-zero systematic bias under domain shift, and strong temporal reproducibility. Importantly, the model showed marked improvements in the pediatric and adolescent interval, where neurodevelopmental nonlinearity and regional asynchrony often induce instability in lifespan regression models. Collectively, these results indicate that coordinated global–local modeling through cross-scale fusion can provide a unified and biologically grounded solution for lifespan-wide brain-age estimation. Future work will focus on extending the framework to multimodal neuroimaging, improving computational efficiency for deployment, enhancing interpretability, and validating clinical utility in disease-specific cohorts.

## Figures and Tables

**Table 1:** Summary of Database and Cohort Characteristics.

Dataset	Age Range	Healthy Participants (n)	Male/ Female	Country	Scanner	Field Strength (T)	Primary Function	Secondary Function	URL
Attention Deficit Hyperactivity Disorder (ADHD200)	7–22	165	81/84	Multiple countries	Multiple scanners	1.5, 3	Unseen Dataset		http://fcon_1000.projects.nitrc.org/ (accessed on 5 June 2024)
Alzheimer’s Disease Neuroimaging Initiative (ADNI)	51–95	506	214/ 292	United States	Multiple scanners	1.5, 3	Internal Dataset		https://adni.loni.usc.edu/ (accessed on 20 July 2024)
age-ility	15–35	131	66/65	Australia	Siemens	3	Internal Dataset		https://www.nitrc.org/projects/age-ility/ (accessed on 12 July 2024)
Brain Genomics Superstruct Project (BGSP)	19–35	1567	661/ 903	United States	Siemens	3	Internal Dataset		https://ida.loni.usc.edu/login.jsp?project=NIFD (accessed on 14 August 2024)
Baby Open Brains Repository (BOBS)	0–1	43	14/29	United States	Siemens	3	Internal Dataset		https://bobsrepository.readthedocs.io/ (accessed on 3 July 2024)
Consortium for Reliability and Reproducibility (CoRR)	6–88	1359	680/ 679	Multiple countries	Multiple scanners	3	Test–Retest Dataset	Internal Dataset	https://doi.org/10.15387/fcp_indi.corr.hnu1 (accessed on 28 August 2024)
Dallas Lifespan Brain Study (DLBS)	20–90	171	52/119	United States	Philips	3	Longitudinal Dataset	Internal Dataset	http://fcon_1000.projects.nitrc.org/indi/retro/dlbs.html (accessed on 5 June 2024)
Frontotemporal Lobar Degeneration Neuroimaging Initiative (NIFD)	36–84	136	58/78	Multiple countries	Multiple scanners	3	Internal Dataset		https://ida.loni.usc.edu/login.jsp?project=NIFD (accessed on 26 August 2024)
Human Connectome Project-adult (HCP-adult)	22–35	85	48/37	United States	Siemens	3	Unseen Dataset		http://www.humanconnectome.org/data/data-use-terms/ (accessed on 14 August 2024)
Human Connectome Project (HCP-retest)	22–38	29	10/19	United States	Siemens	3	Test–Retest Dataset		http://www.humanconnectome.org/data/data-use-terms/ (accessed on 14 August 2024)
Information eXtraction from Images (IXI)	20–87	480	210/ 270	United Kingdom	Multiple scanners	1.5, 3	Unseen Dataset		https://www.nitrc.org/projects/ixi_dataset (accessed on 8 June 2022)
Max Planck Institut Leipzig Mind-Brain-Body Dataset (MPI-LEMON)	20–80	226	145/81	Germany	Siemens	3	Unseen Dataset		http://fcon_1000.projects.nitrc.org/ (accessed on 5 June 2024)
Parkinson’s Disease Datasets	46–82	24	13/11	Multiple countries	Siemens	1.5, 3	Internal Dataset		http://fcon_1000.projects.nitrc.org/ (accessed on 5 June 2024)
Parkinson’s Progression Markers Initiative (PPMI)	32–81	52	30/22	United States	Siemens	3	Unseen Dataset		https://www.ppmi-info.org/ (accessed on 8 June 2022)
Southwest University Adult Lifespan (SALD)	19–80	485	183/ 302	China	Siemens	3	Unseen Dataset		http://fcon_1000.projects.nitrc.org/indi/retro/sald.html (accessed on 5 June 2024)
Southwest University Longitudinal Imaging Multimodal (SLIM)	17–27	573	253/ 320	China	Siemens	3	Internal Dataset		http://fcon_1000.projects.nitrc.org/ (accessed on 5 June 2024)
the Autism Brain Imaging Data Exchang I (ABIDE I)	6–57	366	298/68	Multiple countries	Multiple scanners	3	Internal Dataset		http://fcon_1000.projects.nitrc.org/ (accessed on 5 June 2024)
the Autism Brain Imaging Data Exchang II (ABIDE II)	5–64	461	326/ 135	Multiple countries	Multiple scanners	3	Longitudinal Dataset	Internal Dataset/ Test–Retest Dataset	http://fcon_1000.projects.nitrc.org/ (accessed on 5 June 2024)
The Open Access Series of Imaging Studies I (OASIS I)	18–96	64	17/47	United States	Siemens	1.5	Test–Retest Dataset		https://sites.wustl.edu/oasisbrains/ (accessed on 23 June 2024)
The Open Access Series of Imaging Studies II (OASIS II)	60–96	24	6/18	United States	Siemens	1.5	Test–Retest Dataset		https://sites.wustl.edu/oasisbrains/ (accessed on 23 June 2024)
UK Biobank(UKB)	45–82	16,377	7811/ 8566	United Kingdom	Siemens	3	Internal Dataset		https://www.ukbiobank.ac.uk/ (accessed on 9 June 2020)
Wayne State Study 10 Dataset	19–83	111	35/76	United States	Siemens	1.5	Internal Dataset		http://fcon_1000.projects.nitrc.org/indi/retro/wayne_11.html (accessed on 5 June 2024)
Wayne State Study 11 Dataset	21–79	181	123/58	United States	Siemens	4	Internal Dataset		http://fcon_1000.projects.nitrc.org/indi/retro/wayne_11.html (accessed on 5 June 2024)
Yale High-Resolution Controls Dataset	18–58	120	68/52	United States	Siemens	3	Internal Dataset		http://fcon_1000.projects.nitrc.org/ (accessed on 5 June 2024)
Yale Low-Resolution Controls Dataset	18–66	99	50/49	United States	Siemens	3	Internal Dataset		http://fcon_1000.projects.nitrc.org/ (accessed on 5 June 2024)
Yale Test–Retest Dataset	27–56	12	6/6	United States	Siemens	3	Test–Retest Dataset		http://fcon_1000.projects.nitrc.org/ (accessed on 5 June 2024)
1000 Functional Connectomes Project (FCP1000)	7–85	865	384/ 481	Multiple countries	Multiple scanners	1.5, 3, 4	Internal Dataset/	Test–Retest Dataset/ Longitudinal Dataset	http://fcon_1000.projects.nitrc.org/ (accessed on 5 June 2024)

**Table 2:** Test results and evaluation metrics of different models on the entire internal dataset.

Model Name	MAE	ME	mMAE	r	R^2^
ResNet	4.49	0.27	10.98	0.93	0.86
ViT	4.58	0.01	15.01	0.94	0.89
ResNet-ViT	3.52	−0.41	10.40	0.97	0.94
ResNet-CrossViT	2.72	0.05	5.10	0.98	0.97

**Table 3:** Comparative performance and evaluation metrics across different models for the pediatric and adolescent cohort [0, 18) years within the internal test set.

Model Name	MAE	ME	mMAE	r	R^2^
ResNet	4.83	4.65	7.76	0.52	−1.01
ViT	9.98	9.98	15.01	−0.49	−9.38
ResNet-ViT	4.41	4.36	5.73	0.69	−0.86
ResNet-CrossViT	1.95	1.33	2.12	0.88	0.54

**Table 4:** Comparative performance and evaluation metrics across different models for the adult cohort [18, 60) years within the internal test set.

Model Name	MAE	ME	mMAE	r	R^2^
ResNet	5.88	1.53	10.22	0.87	0.64
ViT	4.93	1.16	10.72	0.93	0.80
ResNet-ViT	3.49	1.22	10.40	0.97	0.90
ResNet-CrossViT	2.60	0.89	5.10	0.98	0.95

**Table 5:** Comparative performance and evaluation metrics across different models for the elderly and senescent cohort [60, 97) years within the internal test set.

Model Name	MAE	ME	mMAE	r	R^2^
ResNet	3.28	−1.01	10.98	0.65	0.27
ViT	3.91	−1.54	12.23	0.46	−0.03
ResNet-ViT	3.47	−1.96	9.60	0.68	0.26
ResNet-CrossViT	2.85	−0.66	4.40	0.78	0.49

**Table 6:** Comparative performance and evaluation metrics across different models on the unseen dataset for cross-domain validation.

Model Name	MAE	ME	mMAE
ResNet	7.15	−3.15	11.35
ViT	7.98	−2.69	15.65
ResNet-ViT	6.37	−0.04	10.67
ResNet-CrossViT	4.19	−0.28	6.40

**Table 7:** Test results and evaluation metrics of different models on the longitudinal dataset.

Model Name	MdE	MAdE	mMAdE
ResNet	−1.83	6.28	11.48
ViT	−1.50	8.86	12.12
ResNet-ViT	−0.53	4.91	9.05
ResNet-CrossViT	−0.50	3.68	6.02

**Table 8:** Test results and evaluation metrics of different models on the retest dataset.

Model Name	σ(y′_scan)	μ(d)	σ(d)	ICC
ResNet	0.76	−0.38	3.51	0.976
ViT	0.48	**−0.12**	2.90	0.982
ResNet-ViT	**0.42**	−0.28	1.94	0.978
ResNet-CrossViT	0.56	−0.51	**1.71**	**0.994**

**Table 9:** Contextual comparison with representative Transformer and Transformer-hybrid brain-age models.

Method	Input	Age Range	Dataset(s)	Sample Size	MAE
Global–Local Transformer [41]	2D MRI slices	0–97	8 public datasets	8379	2.70
Graph Transformer (Brain Networks) [23]	ROI/Graph features	46–81	UKB	16,458	2.71
OpenMAP-BrainAge [42]	3D T1 MRI	42–95	ADNI2&3, OASIS3	2064	3.65
Triamese-ViT [43]	2D views from 3D MRI	6–90	Public MRI datasets	1351	3.87
ResNet-CrossViT (Ours)	3D T1 MRI	0–96	17 public datasets	22,271	2.72

## Data Availability

All data utilized in this study were derived from open neuroimaging databases, with the corresponding access URLs provided in Table 1 of the manuscript. Researchers interested in replicating or extending this work may formally apply for access and subsequent data download through these specified platforms, following the respective institutional guidelines for data retrieval and usage compliance.
